# Transient Elastography-Based Liver Stiffness Age-Dependently Increases in Children

**DOI:** 10.1371/journal.pone.0166683

**Published:** 2016-11-18

**Authors:** Daisuke Tokuhara, Yuki Cho, Haruo Shintaku

**Affiliations:** Department of Pediatrics, Osaka City University Graduate School of Medicine, Osaka, Japan; Università degli Studi di Palermo, ITALY

## Abstract

**Background and Aims:**

Pediatric use of liver transient elastography (TE) is attractive for its non-invasiveness, but reference values have not been established. We aimed to determine reference values for TE in children.

**Methods:**

In pediatric patients (1 to 18 years), TE (FibroScan®) with an M probe was used for both liver stiffness measurement (LSM) and measurement of hepatic fat deposition by using a controlled attenuation parameter (CAP). The patients were divided into three relevant age groups: preschoolers (1 to 5 years), elementary school children (6 to 11 years), and adolescents (12 to 18 years). Overweight or obese patients or those with known liver disease, elevated serum liver enzymes, or hepatic echogenic abnormality were excluded from the study.

**Results:**

Among 139 children, 123 (88.5%; 62 male; median age, 11.7 years; age range, 1.3 to 17.2 years) were successfully subjected to M-probe TE without anesthesia. Median LSM increased with age: it was 3.4 kPa (2.3 to 4.6 kPa, 5th to 95th percentiles) at ages 1 to 5 years; 3.8 (2.5 to 6.1) kPa at ages 6 to 11; and 4.1 (3.3 to 7.9) kPa at ages 12 to 18 (*P* = 0.001). Median CAP was not age dependent: it was 183 (112 to 242) for ages 1 to 18 years.

**Conclusions:**

M-probe TE is suitable in a wide age range of children from age 1 year up. In children without evidence of liver disease, LSM has an age-dependent increase, whereas CAP does not differ between ages 1 and 18.

## Introduction

Liver transient elastography (TE) using FibroScan® (Echosens, Paris, France) is a non-invasive method of assessing liver fibrosis without liver biopsy in both adults and children [1–8]. A recent version of FibroScan was developed for simultaneous liver stiffness measurement (LSM) and measurement of hepatic fat deposition as a controlled attenuation parameter (CAP) [9–11]. Previous studies have demonstrated LSM to be reliable for assessing the severity of various fibrotic liver diseases [1–9] and CAP to be an effective parameter for diagnosing steatosis [9–11]; the combination of LSM and CAP assessment is appropriate for discriminating non-alcoholic steatohepatitis from non-alcoholic fatty liver disease [9,12]. To assess the diagnostic accuracy, it was important to determine cut-off LSM and CAP values for predicting liver fibrosis and steatosis stages and to determine reference values for screening healthy individuals [2–6, 10, 11]. In children, TE is attractive for its non-invasiveness, but firm LSM reference values have not been established because of the limited number of studies [13, 14]. Moreover, to our knowledge, there has been no study focusing on reference CAP values in children. Obesity, metabolic syndrome, gender, and aging are known to affect LSM values in adults [15, 16], and young age should therefore be evaluated as a factor affecting TE values. In this study, we aimed to determine age-dependent reference values for both LSM and CAP in children by using FibroScan® with a 3.5-MHz standard M probe (diameter, 7 mm) designed for simultaneous measurement of LSM and CAP. Although the S probe (5 MHz; diameter, 5 mm) is designed for small children, it can be used only to measure LSM. In addition, a previous study comparing differences in LSM values between the S and M probes demonstrated no significant difference [9], but another study has reported that LSM tends to decrease with increasing probe size [17]. Therefore, it remains controversial as to whether LSM values obtained with an S probe can be considered equivalent to those obtained with the M probe. Consequently, to ensure the same measurement conditions for assessing the age dependency of both LSM and CAP, we used an M probe on all of the pediatric patients in our study. In addition, because general anesthesia has been found to increase LSM values in children [13], we evaluated both LSM and CAP values in children without using general anesthesia.

## Materials and Methods

### Patients

Pediatric patients (1 to 18 years) who received ultrasonographic screening for obesity, proteinuria, urinary tract infection, diarrhea, body weight loss, recurrent abdominal pain, bloody stool, vomiting and liver dysfunction underwent liver TE. We excluded children aged less than 1 year, because our preliminary study had shown that it was difficult to examine these small children by using M-probe TE. Overweight or obese patients (≥90th percentile of body mass index [BMI]), or those with known liver disease, known chromosomal abnormality, elevated serum liver enzyme levels, elevated aspartate aminotransferase (AST) to platelet ratio index (APRI) score (over 0.5), or hepatic echogenic anomaly on abdominal ultrasonography were excluded from the study. Because APRI is a reliable negative predictor of advanced liver disease, we included it in our selection of subjects without liver disease. The patients were divided into three relevant age groups: preschoolers (1 to 5 years), elementary school children (6 to 11 years), and adolescents (12 to 18 years). The Osaka city university graduate school of medicine ethics committee specifically approved this study (the approved number: 2610). In regard to the informed consent, written informed consent was obtained from each child’s parent or legal guardian, and written assent was obtained from children at 6 years old and over, after children and each child’s parent or legal guardian were given, in writing, a full explanation of the aims of the study, its possible hazards, discomfort, and inconvenience. The ethics committee approved this consent procedure.

### Preparation for TE

Before and during the examination, children were allowed to watch popular age-appropriate video programs while lying on the bed beside the TE apparatus. The examiner explained to the children that TE was not painful but was accompanied by a feeling of being pushed. The examiner asked them to be motionless during the examination.

### TE

A skilled examiner who had performed over 500 TE examinations measured LSM and CAP simultaneously by using FibroScan® with the 3.5-MHz standard M probe (diameter, 7 mm). With the patient lying on their back with the right arm in maximum abduction, abdominal ultrasound (AUS) was used to identify a region of the liver that was free of large vascular structures; the tip of the FibroScan® transducer was then placed on the skin between the ribs over the right lobe of the liver, and the LSM value was recorded. The final LSM result was expressed in kilopascals (kPa) and was the median value of 10 individual valid measurements. CAP—a measure of the attenuation of ultrasound waves in the liver at 3.5 MHz—was measured at the same time as LSM by using the M probe. The final CAP value, which ranged from 100 to 400 decibels per meter (dB/m), was the median of 10 individual valid measurements. For each patient, the success rate (SR) was calculated as the ratio of the number of successful measurements to the total number attempted (expressed as a percentage). An examination was considered successful and expressed as measurement completion when 10 valid measurements with a success rate of at least 60% were taken. The measurement completion rate (MCR) was calculated as the ratio of the number of patients with measurement completion to the total number of patients examined by TE. Subjects with unsuccessful examinations were excluded from the analyses.

### Statistical analyses

The three age groups (1 to 5 years, 6 to 11 years, and 12 to 18 years) were compared in terms of patient profile, MCR, SR, examination duration, LSM, and CAP value by using the Kruskal–Wallis test for continuous variables. When the Kruskal–Wallis test identified a significant difference (*P* < 0.05), the Mann–Whitney U test with Bonferroni correction was used to identify the source of the difference. Two-sided *P*-values <0.05 were considered significant. Correlations between age and LSM or CAP were evaluated by using Spearman’s rank correlation coefficient (ρ). Spearman’s ρ values >0.4 were considered indicative of positive correlation.

## Results

### Success of measurement with the M probe

Among 139 children who had no known liver disease, no known chromosomal abnormality, normal serum liver enzyme levels, APRI <0.5, normal ultrasonographic appearance of the liver, and a BMI below the 90th percentile, 123 children (88.5%) (62 male; median age, 11.7 years; age range, 1.3 to 17.2 years) were successfully evaluated by FibroScan (Tables 1 and 2). Data of 107 patients in a total of 139 patients were previously reported as control group in our previous study (9). MCR was significantly lower in preschoolers (76.9%) than in both elementary school children (90.2%, *P* = 0.001) and adolescents (91.9%, *P* < 0.001) (Table 1). There were no significant differences in MCR between elementary school children and adolescents. In preschoolers, the reason for measurement failure was poor cooperation due to excessive motion. SR was significantly lower in preschoolers (86% ± 10%, mean ± SD) than in both elementary schoolers (94% ± 9%, *P* = 0.001) and adolescents (97% ± 6%, *P* < 0.001) (Table 2). There were no significant differences in SR between elementary schoolers and adolescents. Time taken to complete the TE examination was 3.5 ± 3.0 min for all subjects (1 to 18 years), and 2.8 ± 1.1 min for preschoolers, 3.3 ± 2.8 min for elementary schoolers, 4.0 ± 3.5 for adolescents; there were no significant between-group differences (Table 2). These results suggest that FibroScan® measurement using the M probe is feasible for children of a wide range of ages, but that measurement difficulty due to poor cooperation should be taken into consideration in preschoolers.

**Table 1:** Measurement completion rates (MCRs) in 138 children evaluated by transient elastography with an M probe.

	Age (years)			
	Total (1–18)	1–5	6–11	12–18
**Number examined**	139	26	51	62
**Number of measurements completed**	123	20	46	57
**MCR (%)**	88.5	76.9^a^	90.2	91.9

^a^ MCR was significantly lower in children aged 1 to 5 (76.9%) than in children aged 6 to 11 (90.2%, *P* = 0.001) or 12 to 18 (91.9%, *P* < 0.001). There were no significant differences in MCR between children aged 6 to 11 and those aged 12 to 18.

**Table 2:** Profiles and results for 122 children successfully evaluated by transient elastography.

	Age (years)			*P* value, Kruskal–Wallis test	Mann-Whitney U test, *P* value		
	1–5	6–11	12–18		1–5 vs. 6–11	1–5 vs. 10–17	6–11 vs. 10–17
**n**	20	46	57				
**Median age (range)**	4.3 (1.3–5.9)	9.6 (6.0–11.8)	14.0 (12.1–17.2)				
**male**	8	27	27	NS			
**BMI percentile (mean ± SD)**	41.6 ± 29.0	50.7 ± 26.5	38.7 ± 28.5	NS			
**AST, IU/L (mean ± SD)**	30 ± 5	24 ± 5	20 ± 5	<0.001	<0.001	<0.001	<0.001
**ALT, IU/L (mean ± SD)**	13 ± 4	14 ± 6	15 ± 7	NS			
**APRI (mean ± SD)**	0.3 ± 0.1	0.3 ± 0.1	0.2 ± 0.1	0.002	NS	0.004	NS
**SR, % (mean ± SD)**	86 ± 10	94 ± 9	97 ± 6	<0.001	0.001	<0.001	NS
**Exam duration**^**†**^**, min (mean ± SD)**	2.8 ± 1.1	3.3 ± 2.8	4.0 ± 3.5	NS			
**Median LSM, kPa (5th–95th percentiles)**	3.4 (2.3–4.6)	3.8 (2.5–6.1)	4.1 (3.3–7.9)	<0.001	0.04	<0.001	0.04
**Median CAP, dB/m (5th–95th percentiles)**	180 (104–240)	181 (112–240)	190 (111–288)	NS			

ALT, alanine aminotransferase; AST, aspartate aminotransferase; APRI, aspartate aminotransferase to platelet ratio index; BMI, body mass index; CAP, controlled attenuation parameter; LSM, liver stiffness measurement; SR, success rate

^†^ Time taken to complete the transient elastography examination

### Profiles of patients who were successfully examined by FibroScan®

Gender distribution, BMI percentile, and serum ALT level did not differ significantly among age groups (Table 2, S1, S2 and S3 Tables). Serum AST levels and the APRI, which were within the normal ranges in all subjects as a group, were significantly higher in preschoolers than in adolescents (Table 2, S1, S2 and S3 Tables). Because AST is known to show a downward trend with increasing age [18], those statistical differences in AST and APRI between the three age groups are considered as normal difference without any relation to liver involvement.

### LSM reference values

**Fig 1:**
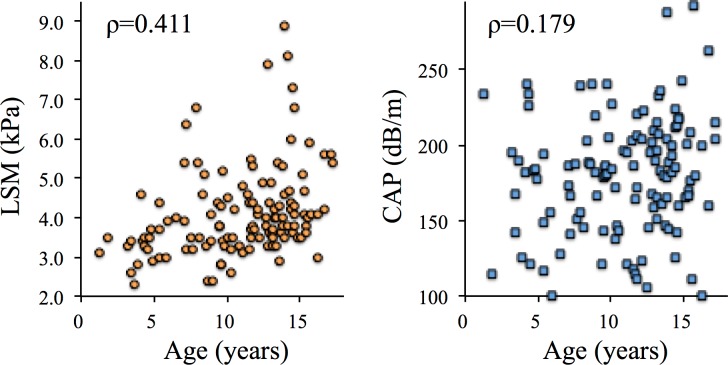
Distributions of liver stiffness measurement (LSM; left) and controlled attenuation parameter (CAP; right) values with age. LSM was positively correlated with age (ρ = 0.411), whereas CAP was not (ρ = 0.179). Correlations between age and LSM or CAP were evaluated by using Spearman’ s rank correlation coefficient (ρ). Spearman’s ρ values >0.4 were considered indicative of a positive correlation.

LSM was positively correlated with age (ρ = 0.411) (Fig 1). Upon categorization of patients into the three age groups, the median LSM showed a significant age-dependent increase, with values of 3.4 kPa (2.3 to 4.6 kPa, 5th to 95th percentile) for preschoolers, 3.8 (2.5 to 6.1) kPa for elementary schoolers, and 4.1 (3.3 to 7.9) kPa for adolescents (Table 2, S1, S2 and S3 Tables). There were no significant differences in LSM between boys and girls in each age group (data not shown).

### CAP reference value

CAP was not positively correlated with age (ρ = 0.179) (Fig 1). Median CAP was not age dependent, with values of 180 dB/m (104 to 240 dB/m, 5th to 95th percentiles) in preschoolers, 181 (112 to 240) dB/m in elementary schoolers, and 190 (111 to 288) dB/m in adolescents (Table 2, S1, S2 and S3 Tables). The median CAP value was 183 (112 to 242) dB/m in children aged 1 to 18 years. There were no significant differences in CAP values between boys and girls in each age group (data not shown).

## Discussion

Here, our results showed age-dependent reference values for LSM and an age-independent reference value for CAP in children over a wide range of ages (1 to 18 years). To date, only two studies have focused on determining normal or reference values of LSM in children over a wide age range [13, 14]. Goldschmidt et al. [13] reported that liver stiffness was not affected by age or gender in children (0 to 18 years) and described the normal median LSM as 4.5 (2.5 to 8.9) kPa in healthy children without notable medical histories, but did not evaluate serum liver enzymes or APRI in their subjects. In contrast, Engelmann et al. [14] reported age-dependent liver stiffness in children. In their study, median values increased with age: they were 4.40 kPa in children aged 0 to 5 years, 4.73 kPa in children aged 6 to 11, and 5.1 kPa in children aged 12 to 18, all of whom were confirmed to have normal serum liver enzymes, normal APRI (<0.5), normal ultrasonographic appearance of the liver, and BMI less than the 90th percentile [14]. We, too, found an age-dependent increase in liver stiffness in children selected as having normal serum liver enzymes, normal APRI, normal ultrasonographic appearance of the liver, BMI less than the 90th percentile, no known liver disease, and no known chromosomal disease—that is, criteria similar to those used by Engelmann et al. These differences in the relationship between age and LSM may reflect natural variations or differences in ethnicity, examination conditions, and sample size, or they may be the result of differences in the approach to probe choice. In the study by Engelmann et al., LSM was assessed in preschoolers and elementary schoolers by using an S or an M probe [14], whereas we measured LSM by using the M probe alone in order to standardize the probe conditions. Because there is controversy as to whether LSM values measured with the S probe are equivalent to those measured with the M probe [9, 13, 17], LSM values obtained by using probes of the same size need to be compared, and reference values need to be set for each type of probe. When we used the M probe for all subjects, we found that LSM was age dependent in children. However, unlike in the study by Engelmann et al. [14], we found no significant differences in LSM between boys and girls. On the basis of our results, we recommend setting reference LSM values according to age, regardless of gender when M probe was used.

In regard to CAP values, to our knowledge there have been no reports demonstrating CAP reference values in children, because the TE used in previous studies has not been able to provide both LSM and CAP values. Here, by using TE providing both CAP and LSM, we revealed that the CAP reference value is age independent in children, and we propose a reference median CAP value of 183 (112 to 242) dB/m for all children aged 1 to 18 years. The reference values indicated here for LSM and CAP will help in the screening of pediatric patients.

Another important point is that we showed that use of the M probe is feasible for children with a wide range of ages from 1 year up. In previous studies, the S probe has been used for preschool and elementary school children [13, 14, 17], but our results showed that use of the M probe is feasible and presents no problems in evaluating both elementary school children and adolescents, possibly because of our effective preparation showing videos, explaining what the procedure would be like in the absence of anesthesia. Measurement difficulties occurred in 23% of preschoolers; nevertheless our results indicated that we can expect high measurement completion rates in preschoolers without anesthesia if patients are prepared mentally and their cooperation obtained. Our results indicated that it takes about 3.5 min to complete a TE examination; it is therefore a key issue for the examiner to keep children motionless for 3 to 5 min. General anesthesia may be unnecessarily invasive for TE screening of relatively healthy children; preparation of children by using video programs and gentle communication appears effective. Because the M probe simultaneously provides CAP and LSM information, its use in preschool and elementary school children may provide new findings in regard to fatty liver disease in this population. In addition, healthcare facilities need to pay USD $35,000 (\4,000,000 in Japan) to purchase an S probe additional to the M probe. The S probe is reportedly appropriate for very small children (with a thorax perimeter of less than 45 cm, which is the average in children aged 1 year [17]); therefore, if a health facility cannot afford both an S probe and an M probe, our results show that it is appropriate to use only the M probe for TE evaluation if the child is aged 1 year or more.

A limitation in this study is the small number of sample to define the reference or normal value of healthy children. In addition, the cut off values of liver stiffness for liver disease are different from the normal range [19]. We clearly showed the age-dependent increase of LSM among the three classes of pediatric age, whereas our results indicated the reference value in those age groups, thus further accumulated study is necessary for the setting of the reference value and cut off values of liver fibrosis.

In conclusion, our study revealed that FibroScan® measurement using an M probe is feasible for children of a wide age range above 1 year, but that measurement difficulties should be taken into consideration in preschool children because of poor cooperation. LSM has an age-dependent increase, whereas CAP does not differ between ages 1 and 18.

## Supporting Information

S1 Table FibroScan and laboratory data of 20 preschoolers. (DOCX)

S2 Table FibroScan and laboratory data of 46 elementary school children. (DOCX)

S3 Table FibroScan and laboratory data of 57 adolescents. (DOCX)

## Abbreviations

TE: transient elastography
LSM: liver stiffness measurement
CAP: controlled attenuation parameter
BMI: body mass index
AST: aspartate aminotransferase
APRI: aspartate aminotransferase-to-platelet ratio index
AUS: abdominal ultrasonography
SR: success rate
MCR: measurement completion rate
7S collagen: type IV collagen 7S
ALT: alanine aminotransferase
kPa: kilopascal
