# Supplementary material for: Transient Elastography-Based Liver Stiffness Age-Dependently Increases in Children
Source: PLoS One. 2016 Nov 18;11(11):e0166683. doi: 10.1371/journal.pone.0166683 (PMC5115769; doi:10.1371/journal.pone.0166683)
Supplement: S1 Table — (DOCX) [file pone.0166683.s001.docx]

| age (yr) | Gender (1=male, 0=female) | Success  Rate  (%) | LSM (kPa) | CAP (dB/m) | BMI percentile | AST  (IU/L) | ALT  (IU/L) | APRI |
| --- | --- | --- | --- | --- | --- | --- | --- | --- |
| 1.3 | 0 | 83 | 3.1 | 234 | 50.7 | 38 | 13 | 0.34 |
| 1.9 | 1 | 83 | 3.5 | 115 | 63.0 | 29 | 10 | 0.27 |
| 3.2 | 0 | 63 | 3.9 | 103 | 20.3 | 30 | 14 | 0.23 |
| 3.2 | 0 | 90 | 3.3 | 195 | 72.4 | 35 | 15 | 0.39 |
| 3.4 | 1 | 77 | 2.6 | 168 | 76.8 | 26 | 12 | 0.28 |
| 3.4 | 0 | 100 | 3.4 | 142 | 4.8 | 34 | 13 | 0.32 |
| 3.6 | 1 | 63 | 2.3 | 190 | 46.9 | 21 | 6 | 0.11 |
| 3.9 | 0 | 89 | 2.8 | 126 | 61.5 | 36 | 12 | 0.36 |
| 4.1 | 1 | 91 | 4.6 | 182 | 5.1 | 32 | 15 | 0.43 |
| 4.2 | 0 | 100 | 3.4 | 240 | 82.2 | 25 | 14 | 0.28 |
| 4.3 | 0 | 91 | 3.5 | 234 | 50.7 | 37 | 23 | 0.42 |
| 4.4 | 1 | 90 | 3.3 | 226 | 62.1 | 29 | 15 | 0.26 |
| 4.5 | 0 | 80 | 3.2 | 121 | 64.6 | 28 | 11 | 0.34 |
| 4.7 | 1 | 91 | 3.5 | 183 | 0.3 | 22 | 11 | 0.33 |
| 4.8 | 0 | 100 | 3.7 | 184 | 64.8 | 34 | 13 | 0.33 |
| 4.9 | 1 | 89 | 2.9 | 177 | 0.2 | 25 | 8 | 0.25 |
| 5.4 | 0 | 77 | 3.0 | 117 | 6.1 | 35 | 14 | 0.47 |
| 5.4 | 1 | 83 | 3.8 | 149 | 44.0 | 36 | 17 | 0.43 |
| 5.4 | 0 | 90 | 3.7 | 194 | 1.6 | 26 | 10 | 0.23 |
| 5.9 | 0 | 83 | 3.0 | 155 | 53.8 | 27 | 13 | 0.27 |
