# Supplementary material for: Transient Elastography-Based Liver Stiffness Age-Dependently Increases in Children
Source: PLoS One. 2016 Nov 18;11(11):e0166683. doi: 10.1371/journal.pone.0166683 (PMC5115769; doi:10.1371/journal.pone.0166683)
Supplement: S2 Table — (DOCX) [file pone.0166683.s002.docx]

| age (yr) | Gender (1=male, 0=female) | Success  Rate  (%) | LSM (kPa) | CAP (dB/m) | BMI percentile | AST  (IU/L) | ALT  (IU/L) | APRI |
| --- | --- | --- | --- | --- | --- | --- | --- | --- |
| 6.0 | 0 | 82 | 3.9 | 100 | 49.4 | 24 | 7 | 0.28 |
| 6.6 | 0 | 71 | 4.0 | 128 | 36.6 | 33 | 11 | 0.33 |
| 7.0 | 1 | 91 | 5.4 | 173 | 71.0 | 23 | 13 | 0.23 |
| 7.1 | 0 | 100 | 3.9 | 186 | 89.5 | 25 | 13 | 0.28 |
| 7.2 | 0 | 100 | 6.4 | 166 | 55.7 | 25 | 13 | 0.33 |
| 7.3 | 1 | 100 | 3.2 | 141 | 82.9 | 27 | 20 | 0.33 |
| 7.3 | 1 | 67 | 2.9 | 183 | 85.0 | 27 | 15 | 0.33 |
| 7.6 | 1 | 100 | 3.5 | 187 | 56.3 | 23 | 11 | 0.28 |
| 7.6 | 1 | 100 | 3.2 | 151 | 15.0 | 32 | 11 | 0.33 |
| 7.9 | 0 | 91 | 6.8 | 239 | 72.9 | 21 | 10 | 0.44 |
| 7.9 | 1 | 100 | 5.4 | 155 | 3.9 | 24 | 8 | 0.28 |
| 8.1 | 0 | 91 | 3.5 | 145 | 45.6 | 21 | 13 | 0.26 |
| 8.4 | 0 | 100 | 4.6 | 203 | 81.4 | 21 | 13 | 0.28 |
| 8.4 | 1 | 100 | 5.1 | 188 | 53.3 | 31 | 15 | 0.29 |
| 8.5 | 0 | 100 | 3.3 | 187 | 63.6 | 27 | 13 | 0.32 |
| 8.6 | 0 | 85 | 2.4 | 240 | 60.9 | 25 | 11 | 0.32 |
| 8.9 | 0 | 100 | 3.4 | 219 | 38.7 | 16 | 11 | 0.13 |
| 9.1 | 0 | 91 | 2.4 | 166 | 66.1 | 28 | 12 | 0.32 |
| 9.3 | 0 | 100 | 4.4 | 121 | 26.4 | 23 | 8 | 0.20 |
| 9.5 | 1 | 100 | 3.8 | 179 | 52.2 | 22 | 10 | 0.34 |
| 9.5 | 0 | 100 | 3.8 | 143 | 31.5 | 30 | 24 | 0.28 |
| 9.6 | 1 | 77 | 4.3 | 185 | 58.1 | 27 | 11 | 0.26 |
| 9.6 | 1 | 100 | 2.8 | 180 | 81.3 | 29 | 27 | 0.29 |
| 9.6 | 0 | 91 | 2.8 | 186 | 76.1 | 16 | 11 | 0.25 |
| 9.7 | 1 | 100 | 5.2 | 240 | 26.3 | 22 | 17 | 0.25 |
| 9.8 | 0 | 77 | 3.4 | 181 | 21.9 | 26 | 21 | 0.30 |
| 9.8 | 1 | 91 | 3.3 | 205 | 88.5 | 22 | 16 | 0.28 |
| 10.0 | 1 | 100 | 4.5 | 227 | 69.6 | 26 | 15 | 0.35 |
| 10.1 | 1 | 100 | 3.5 | 184 | 74.0 | 19 | 11 | 0.13 |
| 10.2 | 1 | 100 | 3.2 | 172 | 79.4 | 16 | 11 | 0.16 |
| 10.3 | 1 | 91 | 2.6 | 138 | 8.2 | 24 | 12 | 0.28 |
| 10.4 | 0 | 100 | 3.5 | 146 | 40.6 | 26 | 13 | 0.32 |
| 10.5 | 1 | 77 | 4.2 | 143 | 20.4 | 28 | 18 | 0.31 |
| 10.9 | 1 | 90 | 3.3 | 196 | 23.5 | 25 | 16 | 0.39 |
| 11.1 | 0 | 91 | 3.1 | 121 | 35.4 | 20 | 12 | 0.29 |
| 11.1 | 1 | 100 | 4.8 | 195 | 85.1 | 23 | 36 | 0.14 |
| 11.4 | 1 | 91 | 4.6 | 203 | 80.3 | 19 | 16 | 0.25 |
| 11.5 | 1 | 100 | 3.2 | 118 | 14.7 | 24 | 14 | 0.36 |
| 11.6 | 1 | 100 | 4.4 | 186 | 85.9 | 13 | 19 | 0.09 |
| 11.7 | 1 | 100 | 5.5 | 164 | 0.0 | 37 | 24 | 0.39 |
| 11.7 | 1 | 100 | 3.5 | 114 | 46.3 | 18 | 6 | 0.18 |
| 11.7 | 0 | 100 | 3.7 | 337 | 15.0 | 21 | 11 | 0.24 |
| 11.7 | 1 | 100 | 5.3 | 172 | 53.4 | 21 | 19 | 0.30 |
| 11.7 | 1 | 83 | 4.4 | 111 | 20.3 | 26 | 15 | 0.24 |
| 11.7 | 1 | 100 | 3.8 | 206 | 20.5 | 21 | 11 | 0.27 |
| 11.8 | 0 | 100 | 3.7 | 221 | 68.5 | 17 | 10 | 0.20 |
