# Supplementary material for: Transient Elastography-Based Liver Stiffness Age-Dependently Increases in Children
Source: PLoS One. 2016 Nov 18;11(11):e0166683. doi: 10.1371/journal.pone.0166683 (PMC5115769; doi:10.1371/journal.pone.0166683)
Supplement: S3 Table — (DOCX) [file pone.0166683.s003.docx]

| age (yr) | Gender (1=male, 0=female) | Success  Rate  (%) | LSM (kPa) | CAP (dB/m) | BMI percentile | AST  (IU/L) | ALT  (IU/L) | APRI |
| --- | --- | --- | --- | --- | --- | --- | --- | --- |
| 12.1 | 0 | 100 | 4.1 | 123 | 13.1 | 25 | 23 | 0.32 |
| 12.1 | 1 | 83 | 4.2 | 204 | 59.8 | 25 | 14 | 0.31 |
| 12.3 | 0 | 100 | 3.5 | 223 | 87.6 | 21 | 21 | 0.28 |
| 12.4 | 0 | 100 | 4.9 | 106 | 12.5 | 19 | 9 | 0.26 |
| 12.6 | 0 | 91 | 4.0 | 145 | 49.7 | 19 | 10 | 0.25 |
| 12.7 | 1 | 100 | 3.8 | 195 | 8.8 | 11 | 6 | 0.10 |
| 12.8 | 0 | 100 | 3.3 | 167 | 2.8 | 23 | 12 | 0.41 |
| 12.8 | 1 | 100 | 7.9 | 202 | 21.7 | 28 | 29 | 0.33 |
| 12.9 | 0 | 100 | 4.4 | 210 | 49.2 | 17 | 9 | 0.21 |
| 13.0 | 0 | 91 | 3.6 | 159 | 83.8 | 17 | 9 | 0.24 |
| 13.1 | 0 | 100 | 4.9 | 190 | 11.2 | 22 | 19 | 0.31 |
| 13.1 | 0 | 91 | 3.6 | 215 | 0.5 | 23 | 13 | 0.25 |
| 13.1 | 1 | 100 | 3.3 | 196 | 8.5 | 27 | 11 | 0.29 |
| 13.2 | 0 | 91 | 3.7 | 151 | 71.0 | 18 | 13 | 0.22 |
| 13.2 | 1 | 100 | 3.5 | 165 | 19.1 | 18 | 7 | 0.22 |
| 13.3 | 0 | 100 | 4.0 | 233 | 18.4 | 13 | 7 | 0.12 |
| 13.3 | 1 | 100 | 4.2 | 207 | 38.5 | 25 | 16 | 0.34 |
| 13.4 | 1 | 91 | 3.3 | 236 | 86.1 | 19 | 31 | 0.20 |
| 13.4 | 0 | 100 | 4.0 | 183 | 54.9 | 19 | 13 | 0.22 |
| 13.5 | 1 | 91 | 5.4 | 303 | 22.3 | 30 | 26 | 0.31 |
| 13.5 | 1 | 100 | 4.3 | 161 | 19.3 | 21 | 13 | 0.33 |
| 13.6 | 0 | 100 | 2.9 | 180 | 33.5 | 14 | 9 | 0.15 |
| 13.7 | 1 | 100 | 3.6 | 146 | 0.2 | 16 | 12 | 0.17 |
| 13.8 | 1 | 100 | 5.3 | 204 | 55.8 | 23 | 18 | 0.34 |
| 13.8 | 1 | 100 | 4.0 | 165 | 5.9 | 19 | 10 | 0.18 |
| 13.8 | 1 | 100 | 3.8 | 179 | 6.6 | 26 | 12 | 0.34 |
| 13.9 | 0 | 100 | 4.6 | 288 | 74.2 | 13 | 8 | 0.11 |
| 13.9 | 0 | 91 | 3.5 | 188 | 88.5 | 17 | 14 | 0.21 |
| 14.0 | 0 | 100 | 8.9 | 144 | 3.5 | 16 | 11 | 0.22 |
| 14.2 | 1 | 100 | 8.1 | 193 | 84.7 | 23 | 9 | 0.25 |
| 14.2 | 0 | 100 | 3.8 | 182 | 4.5 | 15 | 9 | 0.20 |
| 14.2 | 1 | 100 | 4.7 | 200 | 47.8 | 21 | 16 | 0.28 |
| 14.4 | 1 | 100 | 6.0 | 185 | 11.3 | 15 | 19 | 0.11 |
| 14.4 | 0 | 100 | 4.4 | 126 | 8.7 | 21 | 15 | 0.28 |
| 14.4 | 1 | 100 | 4.2 | 224 | 39.8 | 19 | 12 | 0.39 |
| 14.4 | 0 | 100 | 3.6 | 212 | 49.4 | 15 | 7 | 0.12 |
| 14.5 | 1 | 100 | 7.3 | 142 | 53.3 | 23 | 22 | 0.27 |
| 14.6 | 0 | 100 | 4.1 | 213 | 2.6 | 18 | 5 | 0.25 |
| 14.6 | 0 | 83 | 6.8 | 218 | 62.8 | 27 | 30 | 0.44 |
| 14.6 | 0 | 100 | 4.3 | 217 | 52.1 | 16 | 11 | 0.22 |
| 14.7 | 1 | 100 | 3.8 | 160 | 75.7 | 25 | 18 | 0.29 |
| 14.9 | 0 | 83 | 3.5 | 243 | 68.6 | 17 | 13 | 0.18 |
| 15.1 | 0 | 100 | 3.5 | 165 | 48.1 | 18 | 12 | 0.25 |
| 15.2 | 1 | 83 | 5.1 | 201 | 72.8 | 27 | 30 | 0.45 |
| 15.3 | 0 | 100 | 4.1 | 170 | 27.4 | 15 | 8 | 0.25 |
| 15.3 | 1 | 100 | 4.7 | 166 | 47.8 | 17 | 10 | 0.17 |
| 15.4 | 0 | 83 | 3.8 | 176 | 0.1 | 29 | 12 | 0.32 |
| 15.4 | 1 | 100 | 3.6 | 208 | 69.6 | 23 | 14 | 0.26 |
| 15.5 | 0 | 100 | 4.0 | 111 | 42.8 | 14 | 9 | 0.13 |
| 15.7 | 1 | 100 | 5.9 | 292 | 64.5 | 16 | 9 | 0.21 |
| 15.8 | 1 | 100 | 4.1 | 180 | 6.9 | 19 | 12 | 0.25 |
| 16.2 | 0 | 83 | 3.2 | 200 | 59.6 | 23 | 32 | 0.24 |
| 16.2 | 1 | 91 | 4.1 | 100 | 14.3 | 22 | 28 | 0.21 |
| 16.6 | 1 | 100 | 5.6 | 160 | 52.5 | 17 | 13 | 0.22 |
| 16.7 | 1 | 91 | 4.2 | 262 | 87.5 | 15 | 15 | 0.18 |
| 17.1 | 0 | 100 | 5.6 | 204 | 11.3 | 17 | 19 | 0.27 |
| 17.2 | 0 | 100 | 5.4 | 215 | 30.8 | 13 | 13 | 0.22 |
